# PICC tip dislodgement causing massive pleural effusion and atelectasis with acute respiratory failure: a case report

**DOI:** 10.1186/s12887-024-04856-2

**Published:** 2024-07-10

**Authors:** Yao Zhu, Yao Qin, Juan Felipe Alvarez, Wanhong Yin

**Affiliations:** 1 Department of Critical Care Medicine, Zhongnan Hospital of Wuhan University, 169 Donghu Rd, Wuchang District, Wuhan, Hubei Province 430071 China; 2 Department of Critical Care Medicine, West China Hospital of Sichuan University, No.37 Guoxue Alley, Wuhou District, Chengdu, Sichuan Province 610041 China; 3 Department of Cardiology, David Geffen School of Medicine, University of California Los Angeles, Los Angeles, CA 90095 USA

**Keywords:** Pleural effusion, Ultrasound, PICC, Case report

## Abstract

Peripheral intravenous central catheter (PICC) is a common tool for intravenous infusion for children who need central venous access. Although it is safe for physicians and nurses to place, complications like infection, occlusion, phlebitis, and bleeding can occur. We report a 5-month-old infant who suffered respiratory failure caused by catheter malposition resulting in massive fluid infusion into the thoracic cavity. Point-of-care ultrasound (POCUS) was utilized to identify a massive pleural effusion that prompted urgent drainage. Complications related to PICC in pediatric patients are not common but difficult to immediately identify sometimes. Therefore, careful attention should be paid by physicians to identify and reduce the risk of complications associated with PICC. Thus, visual tools are strongly advised to enhance the safety of invasive procedures.

**Supplementary Information:**

The online version contains supplementary material available at 10.1186/s12887-024-04856-2.

## Background

A peripheral intravenous central catheter, serves as a central venous access for children who need short or long-term intravenous infusion or parenteral nutrition and is meaningful for the critically ill. In the pediatric intensive care unit (PICU), peripheral venous access (PVA) is sometimes difficult to establish and cannot fully meet the drug treatment needs of critically ill children. PVA may reduce the efficiency of treatment in children who require infusion of vasoactive drugs, intravenous nutrition, blood products, or intravenous drug bolus at any time. PICC is usually placed through a peripheral vein. Still, in younger children, especially those with lower weight, PICC placement through a peripheral vein may be difficult [1]. The central insertion of PICC catheters is practiced at certain facilities, and there are no specific differences in the management compared to peripherally inserted PICC catheters [2]. There was a risk of bloodstream infection (0.73% vs. 0.24%) [3] for both peripheral and central catheters as an invasive measure. In addition, complications including venous thromboembolism (0.93% vs. 0.52%) [3], catheter blockage, phlebitis, and bleeding were also possible. This report briefly describes a case of acute respiratory failure and massive pleural effusion resulting from catheter misplacement.

## Case presentation

**Fig. 1:**
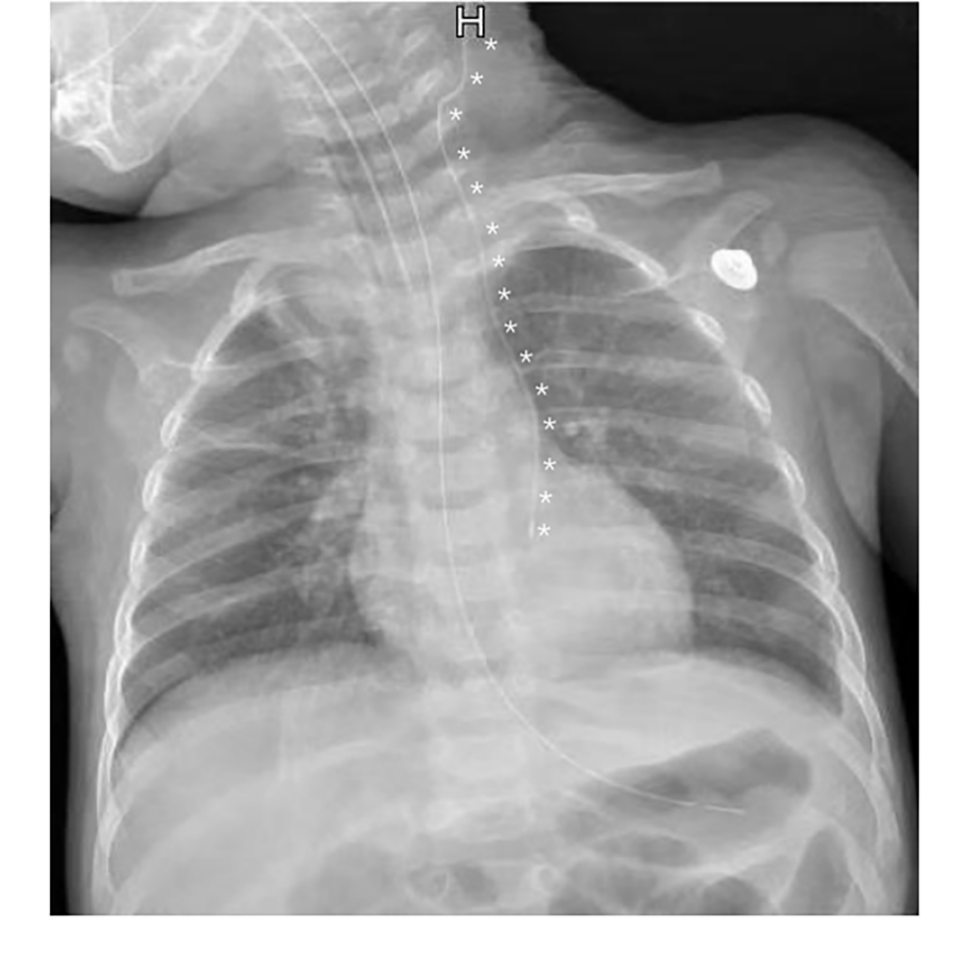
PICC catheter (*), inserted from the left side

**Fig. 2:**
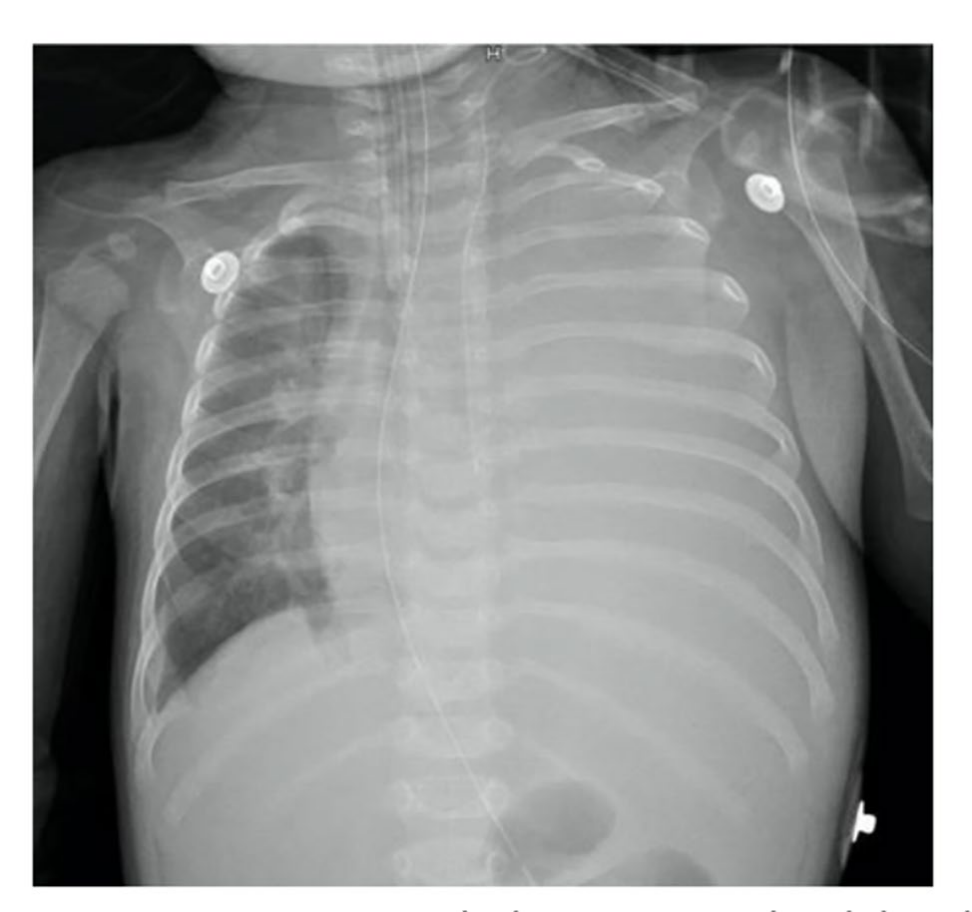
Bedside chest X-ray, engorged thorax and white lung in the left side

**Fig. 3:**
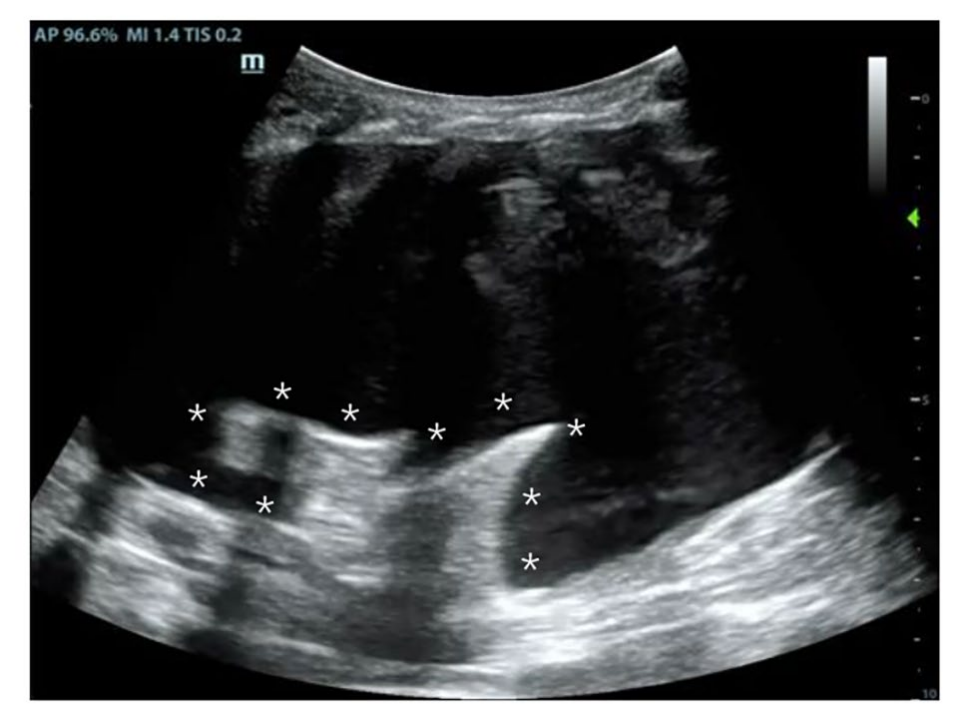
Ultrasound suggested massive pleural effusion, and severe collapsed left lung (*) with “jellyfish” sign

**Fig. 4:**
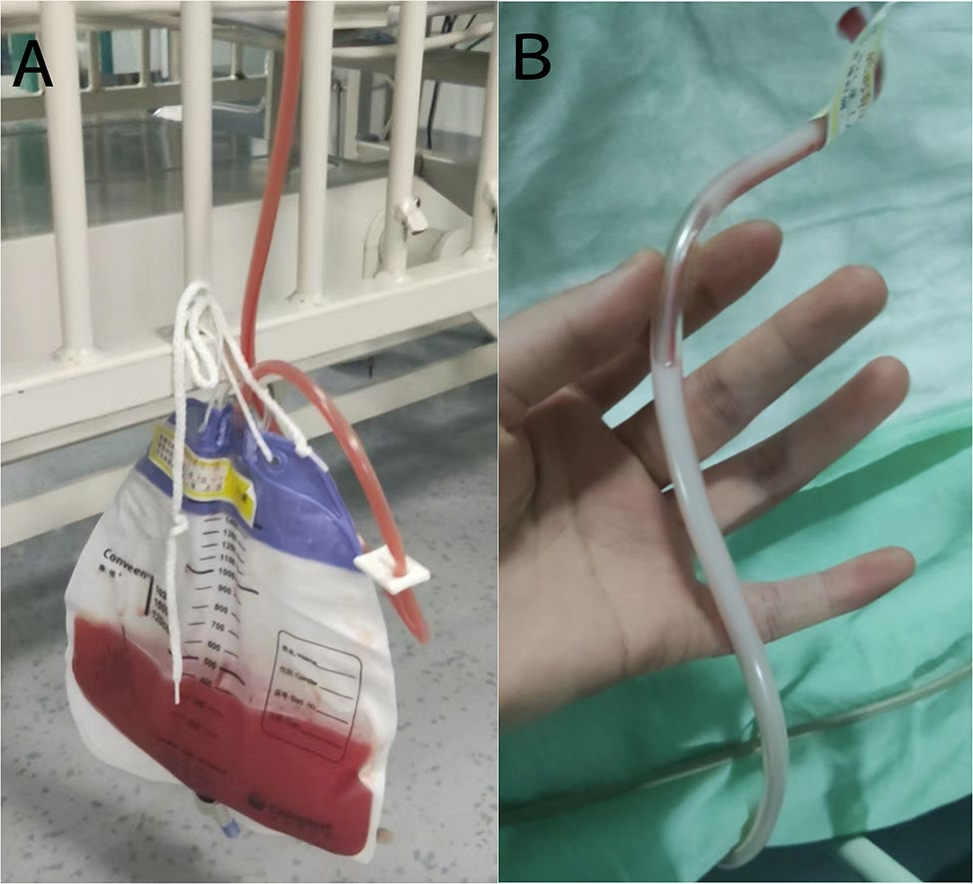
Light bloody liquid in the drainage pack and milky liquid in the tube

A 5-month and 9-day-old male infant was admitted to the pediatric surgery department for crying and vomiting that persisted for 24 h. On the same day, computed tomography (CT) and ultrasonic inspection suggest Epigastric mass and “whirlpool” sign in superior mesenteric vessels. The infant was diagnosed with enteric volvulus and intestinal duplication. After an emergency operation, intestinal obstruction was relieved, and about a 3$\times$4 cm diverticulum-like intestinal canal at a distance of approximately 55 cm from the duodenal flexor ligament against the mesenteric border was cut. There were also no episodes of hypotension, massive bleeding, or other unexpected events during the procedure. The infant was then transferred to PICU to receive postoperative therapy. To give parenteral nutrition (PN), a 3Fr PICC catheter (Bard Access Systems, Inc, Salt Lake City, Utah) was placed in the left internal jugular vein, after being admitted into PICU, guided by ultrasound. A bedside chest X-ray (CXR) (Fig. 1) was performed again to confirm the catheter tip’s location. Considering his condition, supportive treatments like mechanical ventilation, intravenous infusion, sedation, and analgesia were given, and arterial blood gas (ABG) (pH = 7.38, PaO_2_ = 173.2mmHg, PaCO_2_ = 23.7mmHg, BE = -9.61, FiO_2_ = 50%, Hb = 115.1g/L) indicated that his condition was normal. The infant was stable with an oxygenation index greater than 300 on the second day after surgery. After a spontaneous breathing trial, we removed the endotracheal tube. One hour later, the infant showed severe dyspnea with oxygen saturation decreased, ABG (pH = 7.24, PaO_2_ = 72mmHg, PaCO_2_ = 44mmHg, BE = -8.33, FiO_2_ = 50%, Hb = 106.9g/L) suggested acidosis. Following intubation, a bedside CXR was performed, which showed an almost white left lung (Fig. 2). Bedside ultrasonography showed a 26.9 mm liquid dark area with heterogeneous echogenicity (Supplementary file. 1) in the left thoracic cavity, along with the complete collapse of the left lung, showing a typical “jellyfish” sign [4] (Fig. 3). We considered left-sided massive pleural effusion and excluded hemothorax since hemoglobin did not decline consistently. The thoracic puncture was then performed, and approximately 400 mL of light bloody fluid was drained out (Fig. 4). After recruitment maneuvers, milky fluid was drained out, which was propofol infused from PICC. 8 h after the effusion was drained, oxygenation improved consistently. At that time, given the need for continued sedation and analgesia as well as PN, a new PICC was placed.

A bedside CXR within 48 h showed complete drainage of effusion and left lung reexpansion. On the third day after drainage, the endotracheal tube was removed and the infant was transitioned to noninvasive ventilation. The infant was finally transferred to the general ward and discharged successfully.

## Discussion and conclusion

PICC was first used to infuse PN in 1975 [5]. With the development of material science and the expansion of clinical application, it generally became the primary method to infuse critically ill patients. In 1905, Bleichröder applied a central venous catheter (CVC) to the patient for the first time [6] but did not popularize it. In 1949, Duffy used CVC in different types of patients for treatment [7]. Since the invention of PICC, there has been a trend to replace CVC, especially in pediatric critically ill patients and cancer patients undergoing chemotherapy. In some research about PICC, the viewpoint about making CVC out of use is even presented due to the protectiveness, safety, durability, and comfortability of PICC [8–11].

In PICC placement, the modified Seldinger technique is now mostly used for punctures, along with ultrasound-guided placement. Using ultrasound to guide the procedure has been proven necessary for improving the safety and success rate of catheter placement [12, 13]. After PICC is placed, a CXR is often used to ensure the catheter is in the proper position. According to the Infusion Nurses Society (INS) practice standards, The PICC tip should be positioned either in the lower third of the superior vena cava (SVC), the upper third of the right atrium (RA), or near the right cavoatrial junction (CAJ). For lower body insertion catheter, the tip should be positioned in the inferior vena cava (IVC) above the level of the diaphragm [14–16]. In the case presented here, a bedside CXR was performed to affirm the location of the catheter after it was placed. Following operator confirmation, fluid infusion started through PICC. However, the tip of the catheter was positioned in the left thoracic cavity rather than SVC, ultimately causing iatrogenic injury (pleural effusion, atelectasis, and respiratory failure). Tip positioning via fluoroscope of the catheter is an important step in the placement of PICC. For infants and children, CXR positioning is also recommended [17], but it brings radiation damage and may cause long-term health problems [18].

Using the POCUS, intravascular electrocardiogram (ECG), and intravascular Doppler ultrasound can be applied to locate the catheter tip [19, 20]. For atrial fibrillation patients, the accuracy of intravascular ECG is equivalent to CXR [21, 22]. Nevertheless, for medical centers that lack equipment, POCUS can help to locate and also be used to monitor for complications during placement and fluid infusion. When the patient is in the supine position, placing the linear probe on the supraclavicular fossa can scan the long-axis view of bilateral subclavian veins afflux into the SVC and check the location of PICC [23]. Meanwhile, a phased array probe can scan a long-axis view of the IVC, right atrium, and inflowing segment of SVC in the subxiphoid, to check if the tip is in the right position, also avoid PICC being misplaced into the right ventricle to prevent arrhythmia and myocardial lesion [13]. POCUS can also monitor complications like tip dislodgement, accidental dislocation, and pleural effusion caused by dislodgement. In this case, POCUS was used to guide the puncture as well as to identify massive pleural effusion and atelectasis. It assisted in improving the infant’s outcome from acute respiratory failure by tracing the origin of pleural effusion, preventing further injury to the patient.

In addition, proper fixation is an important step to ensure that the catheter and its tip remain in place for a long time after placement. Suture fixation was initially used to ensure the long-term use of the PICC catheter [24]. After the invention of suture-free catheter stabilization devices such as StatLocke device(Bard Access Systems, Inc, Salt Lake City, Utah) and SecurAcath®, many clinical studies have demonstrated that these stabilization devices not only ensure catheter placement compared to suturing, but reduce complications such as catheter-related bloodstream infection (CRBSI), leakage, and displacement [15, 25, 26].

Infants and children are at greater risk for medical emergencies [27–29]. Therefore, when performing invasive procedures, clinicians should pay increased attention to guiding the catheter, avoiding complications, and monitoring patients’ conditions. PICC is widely used in critical pediatric patients, whose complications related to the procedure are not rare. It is highly recommended to utilize POCUS throughout the entire process of PICC management, encompassing puncture and catheterization, confirmation of tip position, as well as monitoring for complications.

## Electronic supplementary material

Below is the link to the electronic supplementary material.

Supplementary Material 1

Supplementary Material 2

## Data Availability

The datasets used and/or analyzed during the case are available from the corresponding author upon reasonable request. If anyone needs data, please contact Wanhong Yin, e-mail: yinwanhong@wchscu.cn.

## Abbreviations

PICC: peripheral intravenous central catheter
PICU: pediatric intensive care unit
PVA: peripheral venous access
CT: computed tomography
PN: parenteral nutrition
ABG: arterial blood gas
CXR: chest X-ray
CVC: central venous catheter
INS: Infusion Nurses Society
SVC: superior vena cava
RA: right atrium
CAJ: cavoatrial junction
IVC: inferior vena cava
POCUS: point-of-care ultrasound
ECG: electrocardiogram
CRBSI: catheter-related bloodstream infection

## References

[1] Bahoush G, Department of Pediatrics, Ali Asghar Children Hospital, Tehran, Iran (the Islamic Republic of), Faculty of Medicine, Iran University of Medical Sciences, Tehran, Iran (the Islamic Republic of). A review of peripherally inserted central catheters and various types of vascular access in very small children and pediatric patients and their potential complications. JMedLife. 2021;14(3):298–309. 10.25122/jml-2020-0011 10.25122/jml-2020-0011 PMC8321608 34377194

[2] Nourzaie R, Abbas H, Parthipun A, et al. Atypical use of PICC as centrally inserted central catheter in infants and neonates: report of a 10-year experience. J Vasc Access. 2023;24(3):409–15. 10.1177/11297298211034308 10.1177/11297298211034308 34320846

[3] Pitiriga V, Bakalis J, Theodoridou K, Kanellopoulos P, Saroglou G, Tsakris A. Lower risk of bloodstream infections for peripherally inserted central catheters compared to central venous catheters in critically ill patients. Antimicrob Resist Infect Control. 2022;11(1):137. 10.1186/s13756-022-01180-1 10.1186/s13756-022-01180-1 PMC9647909 36352414

[4] Han J, Xiang H, Ridley WE, Ridley LJ. Jellyfish sign: pleural effusion. J Med Imaging Radiat Oncol. 2018;62:33–33. 10.1111/1754-9485.20_12785 30309093 10.1111/1754-9485.20_12785

[5] Hoshal VL. Total Intravenous Nutrition with peripherally inserted silicone elastomer central venous catheters. Arch Surg. 1975;110(5):644. 10.1001/archsurg.1975.01360110190032 805577 10.1001/archsurg.1975.01360110190032

[6] Kalso E. A short history of central venous catheterization. Acta Anaesthesiol Scand. 1985;29:7–10. 10.1111/j.1399-6576.1985.tb02313.x 10.1111/j.1399-6576.1985.tb02313.x 3909712

[7] Duffy BJ, the clinical use of polyethylene tubing for intravenous therapy:. A report on seventy-two cases. Ann Surg. 1949;130(5):929–36. 10.1097/00000658-194911000-00008 15393552 10.1097/00000658-194911000-00008 PMC1616349

[8] Fearonce G, Faraklas I, Saffle JR, Cochran A. Peripherally inserted central venous catheters and central venous catheters in burn patients: a comparative review. J Burn Care Res. 2010;31(1):31–5. 10.1097/BCR.0b013e3181cb8eaa 20061834 10.1097/BCR.0b013e3181cb8eaa

[9] Luo X, Guo Y, Yu H, Li S, Yin X. Effectiveness, safety and comfort of StatLock securement for peripherally-inserted central catheters: a systematic review and meta-analysis. Nurs Health Sci. 2017;19(4):403–13. 10.1111/nhs.12361 28730735 10.1111/nhs.12361

[10] Lv Y, Huang X, Lan Y, et al. Peripherally inserted central catheters have a protective role and the effect of fluctuation curve feature in the risk of bloodstream infection compared with central venous catheters: a propensity-adjusted analysis. BMC Infect Dis. 2022;22(1):289. 10.1186/s12879-022-07265-x 35346073 10.1186/s12879-022-07265-x PMC8961920

[11] Yamaguchi RS, Noritomi DT, Degaspare NV, et al. Peripherally inserted central catheters are associated with lower risk of bloodstream infection compared with central venous catheters in paediatric intensive care patients: a propensity-adjusted analysis. Intensive Care Med. 2017;43(8):1097–104. 10.1007/s00134-017-4852-7 28584925 10.1007/s00134-017-4852-7

[12] Al Hamod D, Zeidan S, Al Bizri A, Baaklini G, Nassif Y. Ultrasound-guided central line insertion and standard peripherally inserted catheter placement in preterm infants: comparing results from prospective study in a single-center. North Am J Med Sci. 2016;8(5):205. 10.4103/1947-2714.183011 10.4103/1947-2714.183011 PMC4899959 27298814

[13] Westergaard B, Classen V, Walther-Larsen S. Peripherally inserted central catheters in infants and children - indications, techniques, complications and clinical recommendations: PICCs in children. Acta Anaesthesiol Scand. 2013;57(3):278–87. 10.1111/aas.12024 23252685 10.1111/aas.12024

[14] National Association of Neonatal Nurses. Peripherally inserted central catheters: guideline for practice, 3rd edition[EB/OL]. http://hummingbirdmed.com/wp-content/uploads/NANN15_PICC_Guidelines_FINAL.pdf 10.1097/ANC.0000000000001182 39052577

[15] Waterhouse J, Bandisode V, Brandon D, Olson M, Docherty SL. Evaluation of the Use of a stabilization device to improve the quality of care in patients with peripherally inserted Central catheters. AACN Adv Crit Care. 2014;25(3):213–20. 10.4037/NCI.0000000000000026 25054525 10.1097/NCI.0000000000000026

[16] Nickel B, Gorski L, Kleidon T et al. Infusion Therapy Standards of Practice, 9th Edition. *Journal of Infusion Nursing*. 2024;47(1S):S1-S285. 10.1097/NAN.0000000000000532 10.1097/NAN.0000000000000532 38211609

[17] Evidence-Based Medicine Group, Society N, Chinese Medical Doctor Association. [Operation and management guidelines for peripherally inserted central catheter in neonates (2021)]. Zhongguo Dang Dai Er Ke Za Zhi. 2021;23(3):201–12. 10.7499/j.issn.1008-8830.2101087 33691911 10.7499/j.issn.1008-8830.2101087 PMC7969181

[18] Battiwalla M, Fakhrejahani F, Jain NA, et al. Radiation exposure from diagnostic procedures following allogeneic stem cell transplantation – how much is acceptable? Hematology. 2014;19(5):275–9. 10.1179/1607845413Y.0000000131 24094072 10.1179/1607845413Y.0000000131 PMC4155497

[19] Bidgood C. Improving the patient experience with real-time PICC placement confirmation. Br J Nurs. 2016;25(10):539–43. 10.12968/bjon.2016.25.10.539 27231736 10.12968/bjon.2016.25.10.539

[20] Liu X, Tao X, Xu Y, Zhang X, Chen Y, Wu L. Comparison of bedside ultrasonography and bedside chest radiography in neonatal peripherally inserted central catheters: a before and after self-control study. Front Pediatr. 2022;10:976826. 10.3389/fped.2022.976826 36330366 10.3389/fped.2022.976826 PMC9623023

[21] Albertini F, Struglia M, Faraone V, Fioravanti R, Boursier Niutta S. Effectiveness of the ECG method in the correct positioning of PICC type central venous catheters in patients with atrial fibrillation. Minerva Cardioangiol. 2019;67(3). 10.23736/S0026-4725.19.04915-6 10.23736/S0026-4725.19.04915-6 31116014

[22] Yu C, Shulan L, Juan W, ling L, Chun-Mei L. The accuracy and safety of using the electrocardiogram positioning technique in localizing the peripherally inserted central catheter tip position: a systematic review and meta‐analysis. Nurs Open. 2022;9(3):1556–63. 10.1002/nop2.932 34132498 10.1002/nop2.932 PMC8994971

[23] Criss CN, Claflin J, Ralls MW, Gadepalli SK, Jarboe MD. Obtaining central access in challenging pediatric patients. Pediatr Surg Int. 2018;34(5):529–33. 10.1007/s00383-018-4251-3 29582149 10.1007/s00383-018-4251-3

[24] Graf JM, Newman CD, McPherson ML. Sutured Securement of peripherally inserted Central catheters yields fewer complications in Pediatric patients. J Parenter Enter Nutr. 2006;30(6):532–5. 10.1177/0148607106030006532 10.1177/0148607106030006532 17047181

[25] Frey AM, Schears GJ. Why are we stuck on tape and suture? A review of Catheter Securement devices. J Infus Nurs. 2006;29(1):34–8. 10.1097/00129804-200601000-00007 16428999 10.1097/00129804-200601000-00007

[26] Goossens GA, Grumiaux N, Janssens C, et al. SecurAstaP trial: securement with SecurAcath versus StatLock for peripherally inserted central catheters, a randomised open trial. BMJ Open. 2018;8(2):e016058. 10.1136/bmjopen-2017-016058 29478011 10.1136/bmjopen-2017-016058 PMC5855473

[27] Rea CJ, Alvarez FJ, Tieder JS. The Silent Crisis of Pediatric Clinical Practice guidelines. JAMA Pediatr. 2021;175(12):1201. 10.1001/jamapediatrics.2021.2435 34424270 10.1001/jamapediatrics.2021.2435

[28] Nicolì S, Benevento M, Ferorelli D, Mandarelli G, Solarino B. Little patients, large risks: an overview on patient safety management in pediatrics settings. Front Pediatr. 2022;10:919710. 10.3389/fped.2022.919710 36186651 10.3389/fped.2022.919710 PMC9523149

[29] Lathyris D, Panagiotou OA, Baltogianni M, Ioannidis JPA, Contopoulos-Ioannidis DG. Safety of Medical interventions in Children Versus adults. Pediatrics. 2014;133(3):e666–73. 10.1542/peds.2013-3128 24567023 10.1542/peds.2013-3128 PMC9923602

