# Supplementary material for: PICC tip dislodgement causing massive pleural effusion and atelectasis with acute respiratory failure: a case report
Source: BMC Pediatr. 2024 Jul 10;24:441. doi: 10.1186/s12887-024-04856-2 (PMC11234768; doi:10.1186/s12887-024-04856-2)
Supplement: Supplementary file 1 — Supplementary Material 1 [file 12887_2024_4856_MOESM1_ESM.pdf]

# CARE Checklist – 2016: Information for writing a case report

| Topic | Item | Checklist item description | Line/Page |
|---|---|---|---|
| **Title** | **1** | The words "case report" should be in the title along with the area of focus | 1-2/1 |
| **Key Words** | **2** | Four to seven key words—include "case report" as one of the key words | 3/1 |
| **Abstract** | **3a** | Background: What does this case report add to the medical literature? | 4-6/1 |
| | **3b** | Case summary: chief complaint, diagnoses, interventions, and outcomes | 6-9/1 |
| | **3c** | Conclusion: What is the main "take-away" lesson from this case? | 9-11/1 |
| **Introduction** | **4** | The current standard of care and contributions of this case—with references (1-2 paragraphs) | 12-23/1 |
| **Timeline** | **5** | Information from this case report organized into a timeline (table or figure) | N/A |
| **Patient Information** | **6a** | De-identified demographic and other patient or client specific information | 26/2 |
| | **6b** | Chief complaint—what prompted this visit? | 26-27/2 |
| | **6c** | Relevant history including past interventions and outcomes | 27-32/2 |
| **Physical Exam** | **7** | Relevant physical examination findings | 27-32/2 |
| **Diagnostic Assessment** | **8a** | Evaluations such as surveys, laboratory testing, imaging, etc. | 34/2, 42/2 |
| | **8b** | Diagnostic reasoning including other diagnoses considered and challenges | 28-29/2 |
| | **8c** | Consider tables or figures linking assessment, diagnoses and interventions | 29-32/2 |
| | **8d** | Prognostic characteristics where applicable | 32-33/2 |
| **Interventions** | **9a** | Types such as life-style recommendations, treatments, medications, surgery | 32-48/2-3 |
| | **9b** | Intervention administration such as dosage, frequency and duration | 32-48/2-3 |
| | **9c** | Note changes in intervention with explanation | N/A |
| | **9d** | Other concurrent interventions | N/A |
| **Follow-up and Outcomes** | **10a** | Clinician assessment (and patient or client assessed outcomes when appropriate) | 52-55/3 |
| | **10b** | Important follow-up diagnostic evaluations | 52-55/3 |
| | **10c** | Assessment of intervention adherence and tolerability, including adverse events | N/A |
| **Discussion** | **11a** | Strengths and limitations in your approach to this case | N/A |
| | **11b** | Specify how this case report informs practice or Clinical Practice Guidelines (CPG) | N/A |
| | **11c** | How does this case report suggest a testable hypothesis? | N/A |
| | **11d** | Conclusions and rationale | 91-95/4 |
| **Patient Perspective** | **12** | When appropriate include the assessment of the patient or client on this episode of care | N/A |
| **Informed Consent** | **13** | Informed consent from the person who is the subject of this case report is required by most journals | N/A |
| **Additional Information** | **14** | Acknowledgement section; Competing Interests; IRB approval when required | N/A |
